# Risk factors for Borna disease virus 1 encephalitis in Germany – a case–control study

**DOI:** 10.1080/22221751.2023.2174778

**Published:** 2023-02-27

**Authors:** Kirsten Pörtner, Hendrik Wilking, Christina Frank, Merle M. Böhmer, Klaus Stark, Dennis Tappe

**Affiliations:** a Department of Infectious Disease Epidemiology, Robert Koch Institute, Berlin, Germany; b Postgraduate Training for Applied Epidemiology (PAE), Robert Koch Institute, Berlin, Germany affiliated with the ECDC Fellowship Programme, Field Epidemiology path (EPIET), European Centre for Disease Prevention and Control (ECDC), Solna, Sweden; c Department of Infectious Disease Epidemiology, Bavarian Health and Food Safety Authority, Munich, Germany; d Institute of Social Medicine and Health Systems Research, Otto-von-Guericke-University, Magdeburg, Germany; e Research Group Zoonoses, National Reference Centre for Tropical Pathogens, Bernhard Nocht Institute for Tropical Medicine, Hamburg, Germany

**Keywords:** Bornavirus, BoDV-1, encephalitis, case–control study, risk factors

## Abstract

In 2018, Borna Disease Virus 1 (BoDV-1) was confirmed as a human zoonotic pathogen causing rare but fatal encephalitis in Germany. While diagnostic procedures and the clinical picture have been described, epidemiology remains mysterious. Though endemic areas and a natural reservoir host have been identified with the shrew *Crocidura leucodon* shedding virus in secretions, transmission events, routes and risk factors are unclear. We performed the first comprehensive epidemiological study, combining a large case series with the first case–control study: We interviewed family members of 20 PCR-confirmed BoDV-1 encephalitis cases deceased in 1996–2021 with a standardized questionnaire covering medical history, housing environment, profession, animal contacts, outdoor activities, travel, and nutrition. Cases’ median age was 51 (range 11–79) years, 12/20 were female, and 18/20 lived in the federal state of Bavaria in Southeastern Germany. None had a known relevant pre-existing medical condition. None of the interviews yielded a transmission event such as direct shrew contact, but peridomestic shrew presence was confirmed in 13 cases supporting environmental transmission. Residency in rural areas endemic for animal BoDV-1 was the common denominator of all cases. A subsequent individually matched case–control study revealed residence close to nature in a stand-alone location or on the fringe of the settlement as a risk factor for disease in multivariable analysis with an adjusted OR of 10.8 (95% CI 1.3–89.0). Other variables including keeping cats were not associated with disease. Targeted prevention, future post-exposure-prophylaxis, and timely diagnosis remain challenging.

## Introduction

Borna Disease Virus 1 (BoDV-1; species *Orthobornavirus bornaense*; formerly *Mammalian 1 orthobornavirus*) is an emerging virus characterized by strong neurotropism, and induction of severe encephalitis by a strong T-cell-mediated immunopathogenesis [1–3]. In veterinary medicine, BoDV-1 encephalitis is known as Borna Disease (BD) in mainly horses and sheep and was already described in the eighteenth century [4]. In human medicine BoDV-1 encephalitis remained under the radar for decades. Only in 2018, pathogenicity to humans became apparent in a cluster of solid organ transplant recipients with unclear encephalitis related to a single donor [5] and the simultaneous publication of a sporadic case [6]. Subsequently, around 20 sporadic human BoDV-1-encephalitis cases (acute or identified retrospectively with disease onset going back to 1992) were diagnosed by polymerase chain reaction (PCR) exclusively in Germany and published– all with fatal outcomes [7–14]. Direct pathogen detection of bornaviruses in human samples became mandatorily notifiable in Germany in March 2020. An accompanying nationwide awareness campaign focusing on clinicians, laboratories, and pathologists led to an increase in diagnosed cases [8]. In 2020, a retrospective study could trace back 7/9 previously cryptic fatal encephalitis cases (since 1995) from Bavaria in Southern Germany to BoDV-1 [9], suggesting a significant number of unrecognized cases among unclear fatal encephalitis cases in endemic areas. The only published serological screening for BoDV-1 antibodies among people with partly presumably increased risks (veterinarians and healthy blood donors) predominantly from the endemic area identified only one positive result out of *n* = 1.109 [15] suggesting rareness of the disease and/or an extremely high probability of clinical manifestation and fatality once infected.

The white-toothed bicoloured shrew *Crocidura leucodon* (genus *Crocidura*) is the only known reservoir [16,17], scientific literature on this insectivore shedding the virus in urine, faeces, and saliva [18] is scarce, however. Based on longitudinal veterinary data, endemic areas of BoDV-1 encompass parts of Eastern and Southern Germany and adjacent parts of Switzerland, Austria, and Liechtenstein [1,16,19] while the range of *Crocidura leucodon* reaches from central Europe to Southwest Asia [20,21]. In Germany, the federal state of Bavaria is especially affected by animal BD [15]. Within the endemic area, there are distinct regional genomic sequence clusters as typical for reservoir-bound viruses [1,22].

Whereas diagnostic methods, testing schemes, case definitions, the clinical picture including radiology, and neuropathology have been described [9–11,14,23], the epidemiological characteristics and risk factors for BoDV-1 encephalitis still remain unclear. Particularly the transmission route is puzzling. Human cases are typically already comatose or deceased when BoDV-1 is diagnosed and data on transmission can only be obtained from household members. Also, the interface of the reservoir with humans is completely unknown as *Crocidura leucodon* is a solitary, shy, and nocturnal animal, classified as endangered. Population size is at least temporarily fluctuating [20] while information on synanthropy is rare. So far, only outdoor activities, rural residency, and agricultural work have been hypothesized as risk factors for human infection [9,11] and phylogenetic analyses [9,11] suggest peridomestic infection [8,24]. Reported concrete transmission events would not only inform on the unknown incubation period in humans but also promote diagnosis and allow for potential post-exposure prophylaxis. Knowing the transmission route and risk factors could help formulate preventive measures for a disease characterized by late diagnosis, lack of established therapy, and unfavourable prognosis with short survival times [14].

We describe the first large case series on epidemiological characteristics relying on detailed hypotheses-generating interviews followed by the first case–control study to test hypotheses and possibly guide preventive measures.

## Methods

The case series included acute or retrospectively diagnosed sporadic BoDV-1 cases in Germany. Only PCR-positive cases were included. Recruitment took place from 05/2019 to 07/2022. Preference was given to recently diagnosed cases, minimizing recall bias. Since all cases were already deceased on recruitment, one or two family members per case were interviewed as proxies after giving informed consent mediated by local health authorities or clinicians. To reduce reporting bias, we chose the cases’ closest family members available who had lived for years with the case patient. Family members received written information about the study and the data protection concept. Interviews took place prior to the COVID-19 pandemic at case patients’ homes and during the pandemic by video or telephone call. We used a standardized exploratory, semiqualitative questionnaire covering medical history, details on the case’s place of residence, profession, daily life, outdoor activities, contact to water bodies, travel history, nutrition, recent contact to domestic or wild animals, exposure to ticks and mosquitos, exposure to dust, and potential contacts to shrews and rodents or their secretions. Interviews had to be exploratory with open questions to cover any exposure or activity prior to disease. To assess the interviewees’ awareness of shrews on the premises of the case’s residence, we showed pictures of shrews to family members and/or explained phenotypical differences between shrews and mice. Interviews were performed by at least one epidemiologist and one infectious diseases specialist. Any potential factor relevant to transmission suggested by the interviewees was further explored. Data was pseudonymized, entered in a standardized mask and analysed descriptively. General population age structure (source: Federal Statistical Office of Germany, DESTATIS, census data as of 31 December 2020) was compared to cases’ age distribution using Chi-Square test of independence. Ethical clearance was obtained from the Medical Board of Hamburg (no. PV5616).

Prior to the COVID-19 pandemic, environmental soil samples (*n* = 13) were taken in four cases’ gardens (collected in sheds, along stone walls and other supposed shrew habitats) and analysed for BoDV-1 RNA following a protocol for detection of influenza A virus in soil samples [25]. During the rest of the study period, owing to the COVID-19 pandemic, no further collections were possible.

After 15 interviews and reusing the cases from the case series, we designed an individually matched case–control study based on all 20 cases to investigate the statistical association of BoDV-1 disease with variables such as the living environment, outdoor activities or animal contacts reported. For each case, we recruited 3–5 healthy controls (as statistically beneficial) within the same region of residence (matched by the same 4- to 6-digit land-line telephone area code to yield a close geographic match) and the same age category (11–15, 16–20, 21–30, 31–40, 41–50, 51–60, 61–70, 71–80 years) to adjust for residential and age confounding. To avoid loss of statistical power and introduction of bias resulting from overmatching, we deliberately decided against controls from the same town, neighbourhood or even household. Besides, this would have been against medical confidentiality and EU data protection regulations. To be able to control for confounding by different population sizes of the home communities of cases and controls despite the same telephone area code, we collected the name of the home community and afterwards added the population size (source: Federal Statistical Office of Germany, DESTATIS, census data as of 31 December 2020) in the respective categories (<2.000, 2.000–<5.000, 5.000–<10.000, 10.000–<20.000, 20.000–<50.000, 50.000–<100.000, 100.000–< 500.000, ≥ 500.000 inhabitants). For sensitivity reasons, cases and controls were compared concerning sociodemographics and the matching criteria (sex, age category, population size category of the home community and employment status) using Chi-Square test of independence or Fisher’s exact test as appropriate.

Control interviews were performed in May–June 2022 by telephone by an external market and social research service provider (USUMA; http://www.usuma.com) using another standardized quantitative questionnaire on variables such as the living environment, outdoor activities or animal contacts, especially cats and shrews. Controls were asked about their ability to differentiate shrews from mice and their awareness of the shrew as reservoir host of BoDV-1. Participation was with informed consent as above. Instead of interviewing underage controls, we interviewed minors’ parents. In parallel, the BoDV-1 cases’ data were extracted from the case series’ exploratory semiqualitative interviews and transferred to the controls’ quantitative questionnaire.

To identify risks for disease, we performed univariable and multivariable logistic regression. In the multivariable model we included (i) sex, (ii) the population size of the community of residence as a categorical variable, (iii) the type of housing (living in a detached building), (iv) the relative location of the residence within the settlement (central or marginal), (v) presence of additional structures (sheds, etc.) on the premises, and (vi) whether shrews were being brought on the case’s premises by cats. Variables for the multivariable model were chosen with respect to (statistically significant) association with the outcome in the univariable analysis, effects as potential confounders, plausibility, and the goodness of fit of the model. Sensitivity analyses were performed regarding the matching criteria and the variables included in the multivariable model. Data were analysed using Microsoft Office 2019 Excel and Stata 17.

## Results

### Temporal and geographical distribution of BoDV-1 encephalitis cases

Out of the 35 PCR-confirmed, sporadic BoDV-1 encephalitis cases notified to Robert Koch Institute (RKI) and/or published [6–14,23] by 04/2022 we were able to interview family members of 20 cases, including 90% of eligible cases with onset 2015 through 2021. Family members of 18/20 cases shared the house with the respective case at the time of illness onset. Seven family members were interviewed at cases’ homes, the remaining 13 by video call (*n* = 10) or telephone (*n* = 3). Cases fell ill between 2015 and 2021 except one case in 1996.

**Figure 1.**
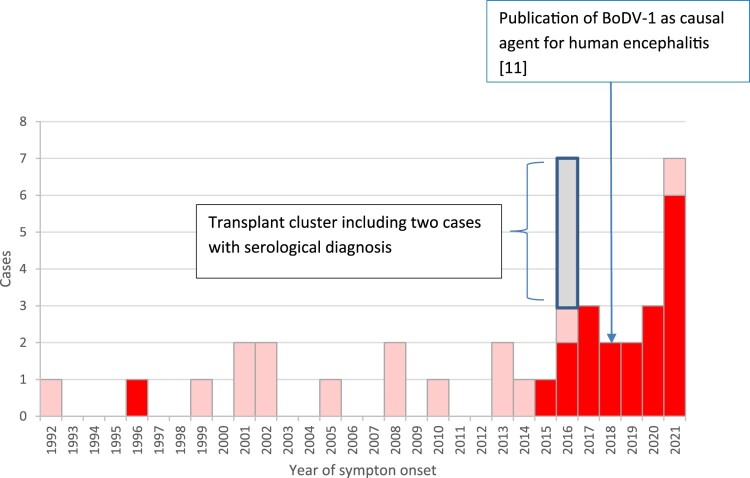
Temporal distribution of published [6–14,23] and/or notified PCR-confirmed sporadic BoDV-1 encephalitis cases in Germany (acutely or retrospectively diagnosed) and the cases of a solid organ transplant cluster (related to a single donor), 1992–2021. Interviewed cases are shown in dark red. Underreporting and missed diagnosis of this rare disease is assumed, especially before the zoonotic potential of BoDV-1 became evident in 2018. Source of notified cases: Robert Koch Institute, Berlin, data as of 04/2022.

**Figure 2.**
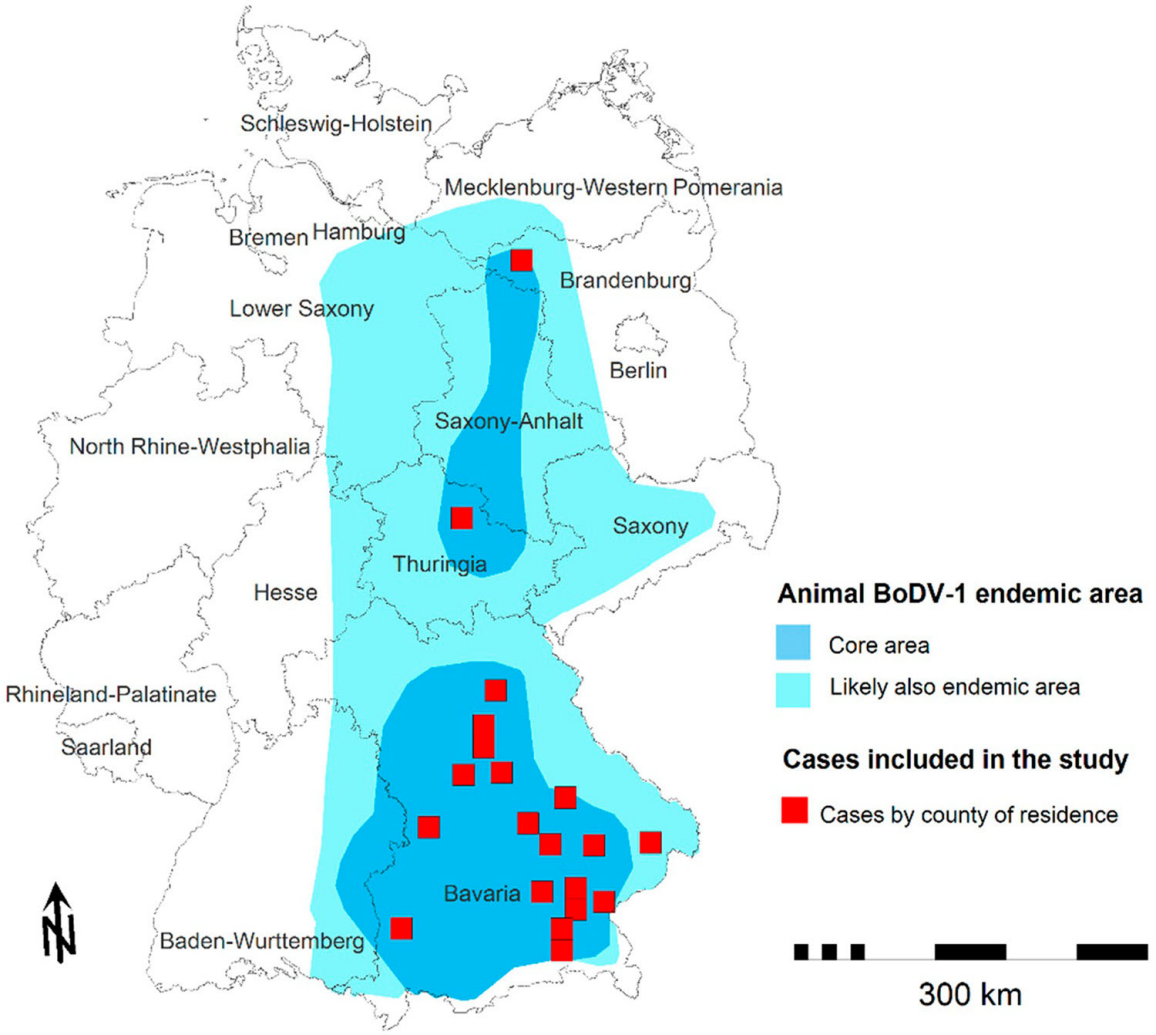
Places of residence of all 20 interviewed cases in Germany deceased in 1996–2021. Each red square equals one case relative to the known endemic area of animal Borna disease (in two shades of blue) [26].

**Figure 3.**
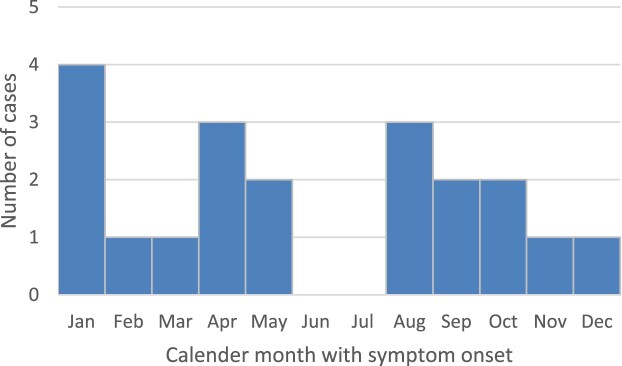
Symptom onset as reported by family members of all 20 interviewed cases by calendar month. So far, a possible seasonality cannot be recognized.

All cases came from the state of Bavaria in Southeastern Germany, except one case each from the states of Brandenburg and Thuringia in Central and Northeastern Germany; temporal or geographic linkage between cases was not apparent. All cases lived in the known animal BD endemic area. Figures 1 and 2 show temporal and geographical distribution of the 20 cases. Symptom onset was widely distributed throughout the calendar year without significant seasonality (Figure 3).

### Demographics of BoDV-1 cases

**Figure 4.**
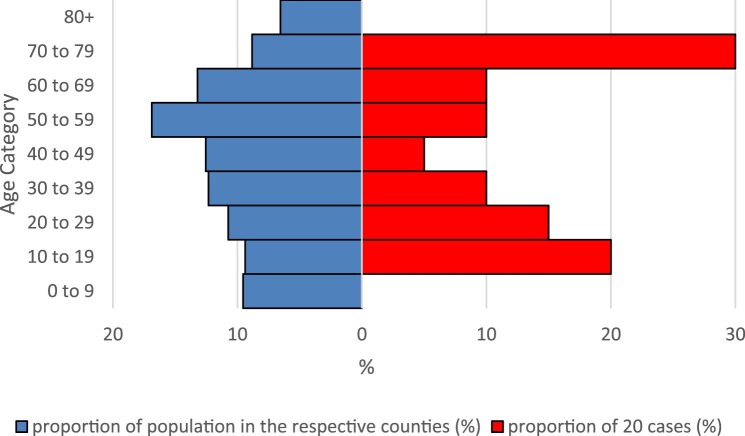
Relative age distribution of BoDV-1 cases (red) and general population (blue) in the counties with cases. Counties with two cases were counted twice. Population data from the Federal Statistical Office of Germany (DESTATIS), census data as of 31 December 2020.

Twelve female and eight male cases made up the 20 cases. Median age was 51 (range 11–79) years. Comparing the age distribution of BoDV-1 cases with that of the general population in the case-affected counties, cases aged 70–79 as well as children and adolescents aged 10–19 and 20–29 appeared overrepresented, while children younger than 10 and elderly persons over 79 were completely absent among the cases (Figure 4). In Chi-Square tests, the proportion of cases in the age category 70–79 years (6/20, 30%) differed significantly from the unaffected population of the respective counties (256,799/2,910,556, 9%, source: Federal Statistical Office of Germany, DESTATIS, census data as of 31 December 2020, *p* = 0.008) compared to the mean of the age categories 20–69 years of cases and the unaffected population, respectively (10% vs. 13%).

Among the cases children and students went to school, working-age adults had jobs and seniors were retired, without apparent exceptions, peculiarities or commonalities.

None of the cases was on immunosuppressive therapy or had a relevant pre-existing medical condition. Within a median of 5.5 weeks after symptom onset (range 3–43), all cases died due to fulminant BoDV-1 encephalitis and/or severe complications.

### Residential characteristics of BoDV-1 cases

Household size included in median two persons (range 1–7). All cases lived very rurally. The most populous affected community (in German “Gemeinde”) of a case had 26.000 inhabitants. Nine cases (45%) lived in communities < 5.000 inhabitants compared to 26% of the Bavarian population and 14% of the German population (source: Federal Statistical Office of Germany, DESTATIS, census data as of 31 December 2020). The households’ locations were frequently on the very fringe of hamlets, villages and two small cities (*n* = 14), five cases lived in an isolated (stand-alone) house outside of settlements (*n* = 5), and only one within the outskirts of a small city (*n* = 1). For the sake of analyses, the latter was regarded as living centrally (neither on the fringe nor isolated). Concerning the type of housing, 18/20 cases lived in a detached house, one in a semi-detached house and one in an apartment building. Eleven out of 19 structures were “modern” buildings (constructed after 1954, according to a German standard) and therefore likely shrew-incursion proof; 12/20 families had noticed at least indirect signs (faeces, signs of gnawing, etc.) of small mammals in the residential building. Additional structures like (former) barns, stables, or sheds existed on 17/20 cases’ premises. All had private gardens on the premises – the case living in the apartment building rented an allotment close by, right on the edge of the settlement. Open land (like meadows, fields, forest, grazing land or uncultivated land) abutted 15/20 cases’ premises. For the other five, the maximum distance from the residence to open land was 200 m (corresponding to the one regarded as living centrally). The next apparent body of water, usually at least a creek, was closer than 500 m to the residential building in 16/20 cases.

**Figure 5.**
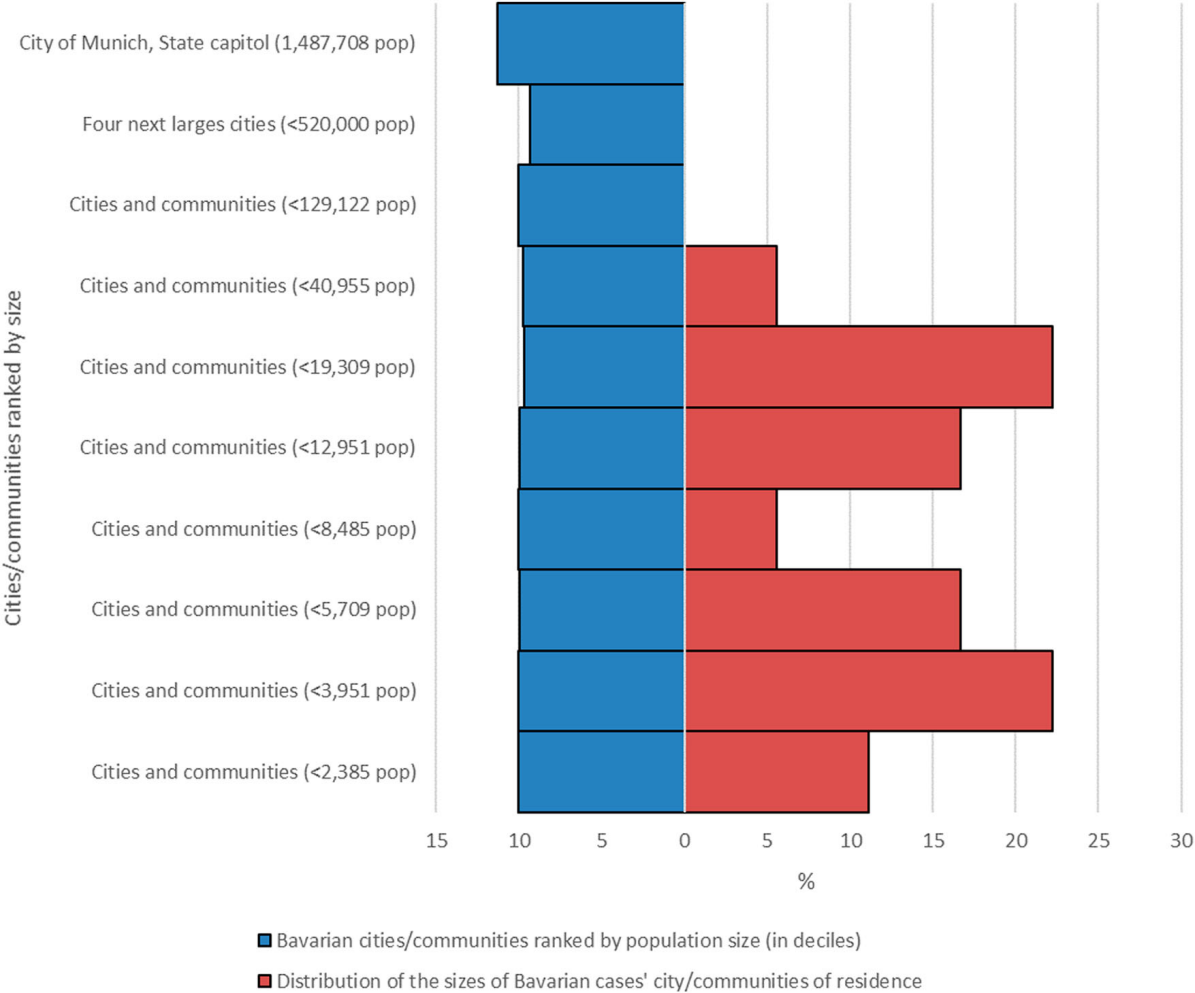
Distribution of the sizes of 18 cases’ cities/communities of residence in the federal state of Bavaria compared to the Bavarian cities/communities ranked by population size (in deciles). Most BoDV-1 encephalitis cases are found in small and very small cities/communities. Population data from the Bavarian Statistical Office, census data as of 31 December 2020.

**Figure 6.**
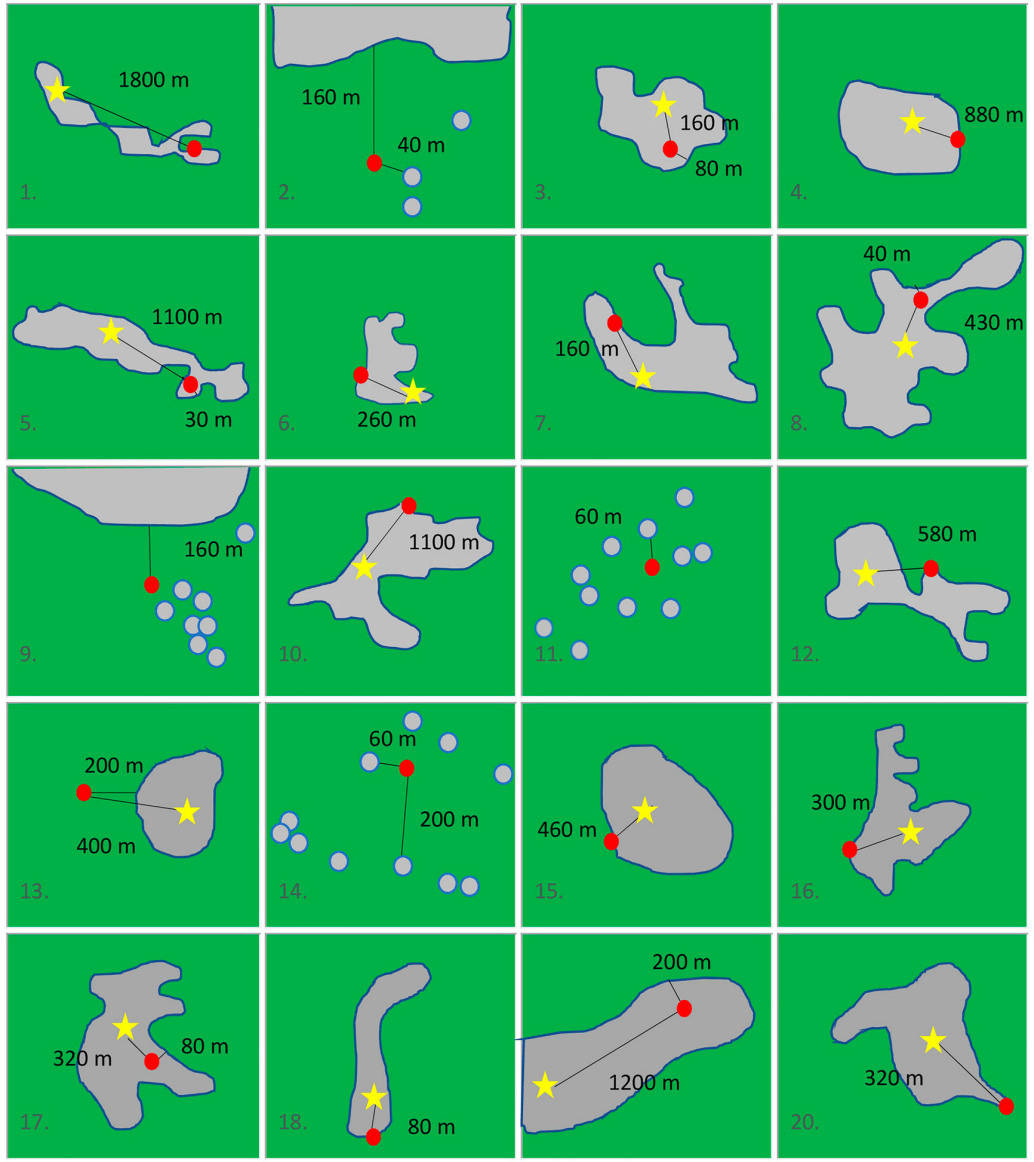
Locations of all 20 cases’ households relative to the centre and the margin of the (closest) built-up area. The red dot shows the respective location of the household, the star the centre of the settlement (defined by the location of the church, commercial clusters etc.), the grey area demarcates the built-up area. Green colour denotes open land. Distances are given in metre. Distances between households (within the built-up area) and the margin of the built-up area are zero metres if not indicated otherwise (panel 1, 4, 6, 7, 10, 12, 15, 16, 18, 20). Panels 2, 9, 11, 13, and 14 represent isolated locations outside of built-up areas whereas all others represent locations on the fringe of the settlement. For the sake of analyses, 19 was regarded as living centrally (neither on the fringe nor isolated).

Figure 5 compares population size distribution of the 18 Bavarian cases’ communities with all Bavarian communities. Case households cluster strongly in small and medium sized communities and were absent from larger cities. Figure 6 shows the schematic locations of all 20 cases’ households relative to the margin and the centre of the closest built-up settlement area (defined by the location of the church, commercial clusters, etc.).

### Exposures

The majority of BoDV-1 cases (14/17) spent much of their spare time outdoors. The outdoor activity most often reported was gardening (14/17). Other, however only individually reported regular activities included jogging, playing soccer, biking, swimming in natural waters, and horseback riding. Home-grown fruits or vegetables were consumed by 17/20 cases and their families. Domestic or farm animals (including cats, dogs, rabbits, sheep, cattle, pigs, horses, fish, lizards, and geese) were kept by 12 case households. Cats were kept by nine households, all of them kept outdoor cats; four others confirmed regular close contact of the case to neighbours’ cats or stray cats. Three cases had relevant animal contact at work, caring for domestic or farm animals.

Nine families reported regular presence of shrews on the premises as within their habitat; six reported cats bringing shrews; altogether, 13 families had noticed shrews in their peridomestic surroundings. Two families reported having had noticed any shrew once within the residential building itself. None of the family members recalled a plausible shrew-related transmission event, such as skin contact, a shrew bite or scratch, the disposal of a carcass or a nest, noticed contact to small mammals’ secretions or shrew infestation of the household.

No apparent commonalities were noted concerning specific outdoor activities (including any outdoor sports, and specifically agriculture, forestry, bathing in natural waters, collecting berries or mushrooms in the forest), profession, nutrition, travelling, contacts with ticks or mosquitos, animal bites, exposure to dust, noticed exposure to small mammals’ faeces, or deep skin injuries. None of the cases had a relevant travel history within or outside of Germany in the months before developing symptoms; half the families reported that the respective case patient had only left the home community for holidays.

### Case–control analysis

Cases and controls differed neither by sex, categorical age, employment status nor the (categorized) population sizes of their home communities.

When comparing rural cases with rural controls, case patients had a 12.3-times (95% CI 1.6–95.2) higher chance to report residence close to nature (stand alone or on the fringe of the settlement) and a 11.0-times (95% CI 1.3–91.5) higher chance to live in a detached building.

In the multivariable model controlling for (i) sex, (ii) the population size of the community of residence, (iii) the type of housing (living in a detached building), (iv) presence of additional structures (sheds, etc.) on the premises, and (v) whether shrews were being brought on the case’s premises by cats the odds ratio for (vi) a residence close to nature was 10.8 (95% CI: 1.3–89.0) (Table 1).

**Table 1.** Results of the case-control analysis with univariable and multivariable logistic regression and individual matching.

			Univariable, individual matching for age category and telephone area code		Multivariable, individual matching for age category and telephone area code	
Variable	Cases (*n* = 20)	Controls (*n* = 80)	Matched OR (95% CI)	*p*-value	Matched aOR (95% CI)	*p*-value
**Age category**						
11–15	3/20 (15%)	12/80 (15%)				
16–20	1/20 (5%)	4/80 (5%)				
21–30	3/20 (15%)	11/80 (14%)				
31–40	2/20 (10%)	7/80 (9%)				
41–50	1/20 (5%)	4/80 (5%)				
51–60	2/20 (10%)	4/80 (5%)				
61–70	3/20 (15%)	16/80 (20%)				
71–80	5/20 (25%)	22/80 (27%)				
**Population of community of residence**^a^			0.9 (0.6–1.4)	0.650	0.8 (0.5–1.5)	0.538
<2.000	1/20 (5%)	12/80 (15%)				
2.000–<5.000	9/20 (45%)	35/80 (44%)				
5.000–10.000	2/20 (10%)	9/80 (11%)				
10.000–<20.000	7/20 (35%)	18/80 (22.5%)				
20.000–<50.000	1/20 (5%)	2/80 (2.5%)				
50.000–<100.000	0/20 (0%)	0/80 (0%)				
100.000–<500.000	0/20 (0%)	2/80 (2.5%)				
≥500.000	0/20 (0%)	2/80 (2.5%)				
**Sex**						
Male	8/20 (40%)	33/80 (41%)	0.9 (0.4–2.6)	0.973	1.1 (0.3–3.7)	0.920
Female	12/20 (60%)	47/80 (59%)				
Employment status: belonging to the working population or going to school	13/20 (65%)	39/80 (49%)	3.2 (0.7–14.4)	0.123		
Year of construction of the residence: built before 1954	8/19 (42%)	23/80 (29%)	1.8 (0.6–5.0)	0.264		
Type of housing: detached building	18/20 (90%)	49/80 (61%)	11.0 (1.3–91.5)	0.026	10.0 (0.9–115.4.)	0.065
Location of the residence within the settlement: close to nature (stand-alone or on the fringe of the settlement)	19/20 (95%)	47/80 (59%)	12.4 (1.6–95.2)	0.016	10.8 (1.3–89.0)	0.027
Garden next to the living building and/or allotment^b^	20/20 (100%)	74/80 (93%)	1.7 (0.2–15.4)^b^	0.648^b^		
Additional structure (shed etc.) on the premises	17/20 (85%)	48/80 (60%)	5.8 (1.2–28.3)	0.031	3.7 (0.6–23.3)	0.160
Open land next to premises	15/20 (75%)	39/79 (49%)	3.6 (1.1–11.7)	0.037		
Touching animals	5/9 (56%)	50/79 (63%)	1.2 (0.3–4.6)	0.810		
Cat/s in the household	9/20 (45%)	31/80 (39%)	1.2 (0.5–3.4)	0.678		
Close contact to cats in general (private or stray)	13/20 (65%)	42/80 (53%)	1.7 (0.6–5.1)	0.321		
Recent skin contact to horses	6/20 (30%)	16/80 (20%)	1.7 (0.5–5.8)	0.391		
Being able to identify a shrew (self-assessment)	13/20 (65%)	63/80 (79%)	0.5 (0.2–1.4)	0.187		
Recent skin contact to shrews^b^	0/20 (0%)	3/63 (5%)	1.2 (0.1–11.1)^b^	0.905^b^		
Shrews living on the premises as within their habitat	9/20 (45%)	24/80 (30%)	1.8 (0.7–4.9)	0.239		
Shrews being brought by cats	6/20 (30%)	11/80 (14%)	2.8 (0.8–9.6)	0.093	2.1 (0.5–8.9)	0.377
Awareness of peridomestic presence of shrews (either as being brought by cats or as having the premises as their habitat)	13/20 (65%)	34/80 (43%)	2.6 (0.9–7.6)	0.074		
Regular outdoor activities	17/20 (85%)	71/80 (89%)	0.7 (0.2–3.0)	0.616		
Specific outdoor activities: gardening	14/17 (82%)	54/80 (68%)	2.9 (0.6–14.7)	0.181		
Spending time outdoors often	14/17 (82%)	53/80 (66%)	2.9 (0.8–11.0)	0.111		
Consuming fruits and/or vegetables from private gardening	17/20 (85%)	53/80 (66%)	2.9 (0.8–10.8)	0.110		
Leaving the home community only for holidays	10/20 (50%)	23/40 (58%)	1.3 (0.4–4.6)	0.721		

Note: Matching was performed for age category and geographical place of residence (telephone area code as a proxy).

OR = odds ratio, aOR = adjusted Odds Ratio, CI = confidence interval.

^a^ Source: Federal Statistical Office of Germany, DESTATIS, census data as of 31 December 2020.

^b^ Convergence could not be achieved with the original data. The data of one case was changed to be able to calculate OR. OR therefore are underestimated.

All other variables were not associated with BoDV-1 disease (including gardening, the only common specific outdoor activity) (Table 1). Especially contact to cats (private or in the neighbourhood), awareness of shrews on the premises or regular outdoor activities in general were not associated with BoDV-1 disease. Since cases did not have any commonalities in specific outdoor activities (other than gardening), an association of individual activities with BoDV-1 encephalitis could not be established.

Sensitivity analyses by excluding two cases and their respective controls with differences in population size of the community of residence (despite matching by the same telephone area code) and by exchanging the shrew-associated variables in the multivariable model showed comparable results.

Awareness of the shrew being the reservoir host of BoDV-1 was low among controls with 60/80 (75%) reporting not to know that the reservoir host of BoDV-1 is the shrew. Among controls (who stated to be able to identify shrews) 3/63 (5%) even confirmed having had recent skin contact to a shrew.

### Environmental samples

No BoDV-1 RNA was detected in the 13 environmental samples of four cases’ gardens. Results are detailed in Table 2 (supplements).

## Discussion

In this study focusing on zoonotic BoDV-1 encephalitis, we investigated for the first time the epidemiological characteristics, and in particular the household environment and living conditions of 20 recent PCR-confirmed fatal human BoDV-1 encephalitis cases. It is the largest case series so far, followed by the first case–control study providing measures of disease association for BoDV-1 encephalitis.

One of the most remarkable results of our study is that not a single plausible transmission event could be found in 20 thoroughly investigated living environments and assessments of exposures. We, therefore, conclude that infections happen covertly for the infected and their household members. This fact also points to difficulties in early diagnosis and defining an incubation period, and will, as far as this becomes possible at some point, also make the indication of post-exposure prophylaxis (or vaccination) very difficult.

Another substantial finding of our study is related to the cases’ very rural residencies. Our study adds to general rurality of all cases’ residencies the more precise finding that cases significantly more often lived on the very fringe of settlements close to nature or even in an isolated location surrounded by open land. This feature of cases’ households, compared to also rural controls, is the strongest and in multivariable analysis the only statistically significant risk factor for BoDV-1 disease with an adjusted OR of 10.8. However, as a risk factor, it largely defies prevention.

Being close to nature with private gardens, meadows, fields, creeks and forests in the direct vicinity generally renders contact with shrews more likely as *Crocidura leucodon* prefers open rural country [20,21]. Additional structures on the premises (even though not a significant variable in the multivariable model), often built from wood and without waterproof floors could serve as a habitat for shrews, providing insect food and shelter. In such spaces, animal secretions are less exposed to harsh weather conditions and BoDV-1 might remain viable for longer periods, increasing the chance of contact with humans.

Supporting the idea of the characteristics of cases’ residencies rendering shrew contacts more probable, shrews were noticed in 9/20 (45%) cases’ surroundings (compared to 24/80 (30%) among controls), and peridomestic shrew presence had an OR of 1.8. However, results were not statistically significant. Shrews are shy animals and may be easily overlooked. On the other hand, shrew population density or BoDV-1 prevalence in shrews might vary. Interestingly, shrews being brought home by cats was not a statistically significant risk factor for infection, either. The hypothesis of the cat bringing home BoDV-1-shedding shrews has been discussed before [15,24,27] as most obvious contact interface. In our study, shrews were brought to cases’ premises regardless of case patients holding cats themselves, such as by stray cats. The cat itself, or contact to cats, was not a risk factor for BoDV-1 disease– likely, because cats are so ubiquitous in rural areas. A differentiation in indoor and outdoor cats was not regarded as being relevant in our study conducted in a very rural setting and therefore not analysed, considering outdoor cats being common in rural settings and indoor cats possibly preying on shrews that have penetrated human habitation. Whether or not cats play a role, either as an intermediate host, a bridge between reservoir host and human being, or as a protective factor by killing shrews therefore remains unclear.

Epidemiological information concerning cases’ behaviour, residential characteristics, shrew presence, and everyday life – including the absence of travel - support the theory of peridomestic infection, our third major finding. Peridomestic transmission possibly takes place either in the house itself (i. e. after the cat bringing shrews into homes or in case of indoor shrew presence), the garden, or the nearby rural surroundings. Results of first phylogenetic studies with case patients’ virus sequences clustering with local animal and human sequences [5,9,11] provide further evidence on local, peridomestic zoonotic transmission events.

Indirect, environmental transmission via the shrews’ secretions appears likely since direct shrew contact was not reported. The first environmental samples to be analysed for BoDV-1 showed negative results in our study, but our sample size is limited. Extensive soil analysis should be performed in the future complemented by studies on the viral tenacity.

Notably, our data suggest that some other commonly suggested factors are not associated with BoDV-1 infection. This includes contacts to other animals, either domestic or agricultural animals, general outdoor activities, or consuming home-grown products*.* Even larger case series might not be able to show an association of certain factors (such as specific outdoor activities) and BoDV-1 disease; transmission appears to be environmental and the virus is likely unevenly distributed.

Our data does not suggest a sex preference and there is no obvious predisposing medical condition. Cases predominating in the elderly and the young may be due to more frequent/lengthy outdoor exposures compared to working-age adults.

The strength of our study is its epidemiological in-depth analysis and its two-step design: a detailed case series followed by a case–control study. The matching criteria very well respected the peculiarities of the endemic area and very rural residency seen in the cases. At the same time, we avoided overmatching by not matching for the same town, neighbourhood or even household. While the small absolute sample size is a limitation, given the low incidence, the size is relatively large. Nevertheless, the small sample size and the overall limited knowledge of pathogen and reservoir might lead to underestimation of particular risk factors.

Our study shows that BoDV-1-awareness of people living in endemic regions is low, even in communities in the close neighbourhood of a case. A first step to prevent this fatal viral zoonosis requires a better understanding of the endemic areas or epidemiological hotspots [16] based on comprehensive human and animal data. Data on shrew populations, shrew behaviour and shrew colonization is needed. Expanded pet and domestic animal seroprevalence studies as well as BoDV-1 detection in wild shrews and comprehensive phylogenetic analyses may provide more insight into the distribution of BoDV-1 in certain geographic areas such as close to patients’ homes.

A second step could be a systematical health education of the general population, especially in endemic areas, to at least reduce exposures. Shrews have to be perceived as reservoir hosts by the population in endemic areas, antagonized (by withdrawing the food source for example) and handled- if at all – with at least personal protective equipment like gloves and masks. Our finding of 5% of controls confirming to have had recent skin contact to a shrew underlines the unawareness of shrew-associated risks. The possibility of area-wide antagonizing of *Crocidura leucodon*, a protected species, needs to be discussed by the respective experts and authorities.

A long and maybe variable incubation period could obscure seasonality in this case series. In animal disease peaks in spring and summer, presumably caused by shrews entering stables of domestic or agricultural animals in the need for shelter during the winter before, have been observed and discussed [16,28]. This could apply for human residencies as well since *Crocidura leucodon* is known to be able to enter human habitations [20,29], and larger case series might be able to show seasonality in the future. For the virus to enter the human body, minor skin lesions, the oro-nasal mucosa (alternatively by inhalation of contaminated dust or ingestion of contaminated fruit and vegetables) cannot be excluded. The olfactory route is described for horses during grazing [30]. To shed more light on possible infection routes, animal models, environmental studies, and studies on viral tenacity are needed.

The apparent lack of an obvious transmission event, possibly multiple transmission routes, no discernable seasonality, a possibly lengthy variable incubation period along with low incidence and lack of reported local clusters may explain why this fateful, fatal disease remained undetected for decades in human medicine. Further investigations of additional cases may provide clues to these unresolved epidemiological questions of human BoDV-1 encephalitis.

## Supplementary Material

Supplemental Material

## References

[1] Rubbenstroth DSK, Schwemmle M, Rissland J, et al. Human bornavirus research: back on track!. PLoS Pathog. 2019;15(8):e1007873. 31369648 10.1371/journal.ppat.1007873 PMC6675037

[2] Richt JA, Rott R. Borna disease virus: a mystery as an emerging zoonotic pathogen. Vet J. 2001 Jan;161(1):24–40. 11145828 10.1053/tvjl.2000.0533

[3] Lipkin WI, Briese T, Hornig M. Borna disease virus - fact and fantasy. Virus Res. 2011 Dec;162(1-2):162–172. 21968299 10.1016/j.virusres.2011.09.036

[4] Dürrwald R, Ludwig H. Borna disease virus (BDV), a (zoonotic?) worldwide pathogen. A review of the history of the disease and the virus infection with comprehensive bibliography. Zentralbl Veterinarmed B. 1997 May;44(3):147–184. 9197210 10.1111/j.1439-0450.1997.tb00962.x

[5] Schlottau K, Forth L, Angstwurm K, et al. Fatal encephalitic Borna disease virus 1 in solid-organ transplant recipients. N Engl J Med. 2018 Oct 4;379(14):1377–1379. 30281984 10.1056/NEJMc1803115

[6] Korn K, Coras R, Bobinger T, et al. Fatal encephalitis associated with Borna disease virus 1. N Engl J Med. 2018 Oct 4;379(14):1375–1377. 30281979 10.1056/NEJMc1800724

[7] Coras R, Korn K, Kuerten S. Severe bornavirus-encephalitis presenting as Guillain–Barré-syndrome. Acta Neuropathol. 2019;137:1017–1019. 30953131 10.1007/s00401-019-02005-z

[8] Frank C, Wickel J, Bramer D, et al. Human Borna disease virus 1 (BoDV-1) encephalitis cases in the north and east of Germany. Emerg Microbes Infect. 2022 Dec;11(1):6–13. 34783638 10.1080/22221751.2021.2007737 PMC8725967

[9] Niller HH, Angstwurm K, Rubbenstroth D, et al. Zoonotic spillover infections with Borna disease virus 1 leading to fatal human encephalitis, 1999-2019: an epidemiological investigation. Lancet Infect Dis. 2020 Apr;20(4):467–477. 31924550 10.1016/S1473-3099(19)30546-8

[10] Liesche F, Ruf V, Zoubaa S, et al. The neuropathology of fatal encephalomyelitis in human Borna virus infection. Acta Neuropathol. 2019 Oct;138(4):653–665. 31346692 10.1007/s00401-019-02047-3 PMC6778062

[11] Eisermann P, Rubbenstroth D, Cadar D, et al. Active case finding of current bornavirus infections in human encephalitis cases of unknown etiology, Germany, 2018-2020. Emerg Infect Dis. 2021 May;27(5):1371–1379. 33900167 10.3201/eid2705.204490 PMC8084505

[12] Tappe D, Pörtner K, Frank C, et al. Investigation of fatal human Borna disease virus 1 encephalitis outside the previously known area for human cases, Brandenburg, Germany - a case report. BMC Infect Dis. 2021 Aug 10;21(1):787. 34376142 10.1186/s12879-021-06439-3 PMC8353434

[13] Meier H, Bauer C, Finkenzeller W. Die Borna-Virus-Enzephalitis als Differenzialdiagnose zur seronegativen Autoimmunenzephalitis [Bornavirus-encephalitis as a differential diagnosis of seronegative autoimmune encephalitis]. Nervenarzt. 2022;93:835–837. German. 35024881 10.1007/s00115-021-01259-x PMC8756745

[14] Neumann B, Angstwurm K, Linker RA, et al. Antibodies against viral nucleo-, phospho-, and X protein contribute to serological diagnosis of fatal Borna disease virus 1 infections. Cell Rep Med. 2022 Jan 18;3(1):100499. 35106511 10.1016/j.xcrm.2021.100499 PMC8784767

[15] Tappe D, Frank C, Offergeld R, et al. Low prevalence of Borna disease virus 1 (BoDV-1) IgG antibodies in humans from areas endemic for animal Borna disease of southern Germany. Sci Rep. 2019 Dec 27;9(1):20154. 31882942 10.1038/s41598-019-56839-4 PMC6934520

[16] Dürrwald R, Kolodziejek J, Weissenbock H, et al. The bicolored white-toothed shrew *Crocidura leucodon* (HERMANN 1780) is an indigenous host of mammalian Borna disease virus. PLoS One. 2014;9(4):e93659. 24699636 10.1371/journal.pone.0093659 PMC3974811

[17] Hilbe M, Herrsche R, Kolodziejek J, et al. Shrews as reservoir hosts of Borna disease virus. Emerg Infect Dis. 2006 Apr;12(4):675–677. 16704819 10.3201/eid1204.051418 PMC3294707

[18] Nobach D, Bourg M, Herzog S, et al. Shedding of infectious Borna disease virus-1 in living bicolored white-toothed shrews. PLoS One. 2015;10(8):e0137018. 26313904 10.1371/journal.pone.0137018 PMC4552160

[19] Weissenbock H, Bago Z, Kolodziejek J, et al. Infections of horses and shrews with bornaviruses in upper Austria: a novel endemic area of Borna disease. Emerg Microbes Infect. 2017 Jun 21;6(6):e52. 28634359 10.1038/emi.2017.36 PMC5520313

[20] Mitchell-Jones A, Amori G, Bogdavnovicz W. The atlas of European mammals (Poyser natural history). 1st ed. Cambridge: Acadamic Press; 1999.

[21] Shenbrot G, Hutterer R, Kryštufek B, et al. *Crocidura leucodon* (amended version of 2016 assessment). The IUCN Red List of Threatened Species 2021 2021. Available from: https://www.iucnredlist.org/species/29651/197500630#amendment.

[22] Kolodziejek J, Dürrwald R, Herzog S, et al. Genetic clustering of Borna disease virus natural animal isolates, laboratory and vaccine strains strongly reflects their regional geographical origin. J Gen Virol. 2005 Feb;86(Pt 2):385–398. 15659758 10.1099/vir.0.80587-0

[23] Finck T, Liesche-Starnecker F, Probst M, et al. Bornavirus encephalitis shows a characteristic magnetic resonance phenotype in humans. Ann Neurol. 2020 Oct;88(4):723–735. 32794235 10.1002/ana.25873

[24] Meyer T, Tappe D, Hasan D. “Borna disease virus 1” (BoDV-1)-Enzephalitis eines 18-Jährigen außerhalb des bisher bekannten Endemiegebietes [Borna disease virus 1 (BoDV-1) encephalitis in an 18-year old outside the known endemic area]. DGNeurologie. 2022;5:300–304. German.

[25] Deboosere N, Horm SV, Delobel A, et al. Viral elution and concentration method for detection of influenza A viruses in mud by real-time RT-PCR. J Virol Methods. 2012 Jan;179(1):148–153. 22036660 10.1016/j.jviromet.2011.10.013

[26] Pörtner K, Wilking H. Informationen zur Vermeidung von Infektionen mit dem Borna Disease Virus 1 [Information on preventing infections due to Borna Disease Virus 1]: Robert Koch Institute; 2019. Available from: https://www.rki.de/DE/Content/InfAZ/B/Bornavirus/Merkblatt.pdf?__blob=publicationFile.

[27] Pörtner K, Frank C, Schmidt-Chanasit J, et al. Bornavirus-Infektionen: Hohe Letalität durch fulminante meningoenzephalitiden [Bornavirus-infections: fulminant meningoencephalitis with high fatality]. Dtsch Arztebl. 2019;116(50):A-2350–2354. German.

[28] Dürrwald R, Kolodziejek J, Muluneh A, et al. Epidemiological pattern of classical Borna disease and regional genetic clustering of Borna disease viruses point towards the existence of to-date unknown endemic reservoir host populations. Microbes Infect. 2006 Mar;8(3):917–929. 16469519 10.1016/j.micinf.2005.08.013

[29] Kraft R. Mäuse und Spitzmäuse in Bayern: Verbreitung, Lebensraum, Bestandssituation [Mice and shrews in Bavaria. Geographic range, habitat, population trend.]. 1st ed. Stuttgart: Verlag Eugen Ulmer; 2008.

[30] Kupke A, Becker S, Wewetzer K, et al. Intranasal Borna disease virus (BoDV-1) infection: insights into initial steps and potential contagiosity. Int J Mol Sci. 2019 Mar 15;20(6):1318. 30875911 10.3390/ijms20061318 PMC6470550

