# Supplementary material for: Risk factors for Borna disease virus 1 encephalitis in Germany – a case–control study
Source: Emerg Microbes Infect. 2023 Feb 27;12(1):e2174778. doi: 10.1080/22221751.2023.2174778 (PMC9980402; doi:10.1080/22221751.2023.2174778)
Supplement: Supplemental Material [file TEMI_A_2174778_SM5102.docx]

| **Case** | **Sample No.** | **Location** | **Result** |
| --- | --- | --- | --- |
| A | 1 | soil and grass clippings | Negative |
|  | 2 | soil hedgehog shed | Negative |
|  | 3 | sand with faeces from sandbox | Negative |
|  | 4 | mulch near garden wall | Negative |
|  | 5 | mulch near currant hedge | Negative |
|  | 6 | playground soil | Negative |
| B | 7 | dirt in woodworking shop | Negative |
|  | 8 | faeces and dirt on terrace | Negative |
|  | 9 | garden bed | Negative |
| C | 10 | garden compost | Negative |
|  | 11 | stone wall dirt | Negative |
|  | 12 | stone wall dirt | Negative |
| D | 13 | garden compost | Negative |

**Table 2. Details on 13 soil samples of four cases (A-D) concerning location and result.** Environmental soil samples (n=13) were taken in four cases´ gardens (collected in sheds, along stone walls and other supposed shrew habitats) and analyzed for BoDV-1 RNA following a protocol for detection of influenza A virus in soil samples [25]. Results were negative**.**
